# PReS-FINAL-2150: Antiocular antibodies in children with juvenile idiopathic arthritis-associated uveitis

**DOI:** 10.1186/1546-0096-11-S2-P162

**Published:** 2013-12-05

**Authors:** K Walscheid, M Hennig, C Heinz, D Bauer, M Dietzel, M Busch, S Wasmuth, D Foell, C Deeg, A Heiligenhaus

**Affiliations:** 1 Department of Ophthalmology, St. Franziskus-Hospital Muenster, Muenster, Netherlands; 2 Department of Pediatric Rheumatology and Immunology, University Hospital Muenster, Muenster, Netherlands; 3 Ophtha-Lab; Department of Ophthalmology, St. Franziskus-Hospital Muenster, Muenster, Netherlands; 4 Department of Animal Physiology, Ludwig Maximilians University Munich, Munich, Germany

## Introduction

Juvenile idiopathic arthritis (JIA) is the most common disease associated with uveitis of childhood. The pathogenesis of JIA-associated uveitis (JIAU) is undefined, although there is evidence for a B-cell-mediated autoimmune process with a probably pathogenetic role for autoantibodies.

## Objectives

This study intended to analyze the antiocular autoantibodies in serum and their correlation with disease course.

## Methods

Serum samples from children with JIAU (n = 47), JIA without uveitis (n = 67), idiopathic anterior uveitis (IAU, n = 12) and healthy controls (n = 52) were collected. The binding patterns of serum antibodies to ocular cryosections from swine eyes were analyzed by indirect immunohistochemistry, and were correlated to epidemiological, clinical and laboratory test results.

## Results

The patient groups differed with respect to their presence of antibody-binding to the sections: JIAU (94%), JIA (75%), IAU (75%), and healthy controls (29%) to uveal and/or retinal structures. Serum antibodies of JIAU patients predominantly bound at iris (74%) and ciliary body (cb, 79%). Iris/cb positive staining correlated with the presence of uveitis complications (p < 0.005) in JIAU patients, but not with positivity for serum anti-nuclear antibodies (ANA), rheumatoid factor (RF) or HLA-B27, and was independent from uveitis activity or type of anti-inflammatory treatment.

## Conclusion

In JIAU patients, anti-ocular serum antibodies can be detected more frequently than in control groups. Binding patterns to ocular tissue correlate with complicated uveitis course but not with uveitis activity and anti-inflammatory treatment. Antibody-binding is not specific for this uveitis entity, and does not correlate with ANA-positivity.

## Disclosure of interest

None declared.

